# Uncovering missed indels by leveraging unmapped reads

**DOI:** 10.1038/s41598-019-47405-z

**Published:** 2019-07-31

**Authors:** Mohammad Shabbir Hasan, Xiaowei Wu, Liqing Zhang

**Affiliations:** 1 Department of Computer Science, Virginia Tech, Blacksburg, VA 24061 USA; 2 Department of Statistics, Virginia Tech, Blacksburg, VA 24061 USA

**Keywords:** Software, Genomics

## Abstract

In current practice, Next Generation Sequencing (NGS) applications start with mapping/aligning short reads to the reference genome, with the aim of identifying genetic variants. Although existing alignment tools have shown great accuracy in mapping short reads to the reference genome, a significant number of short reads still remain unmapped and are often excluded from downstream analyses thereby causing nonnegligible information loss in the subsequent variant calling procedure. This paper describes Genesis-indel, a computational pipeline that explores the unmapped reads to identify novel indels that are initially missed in the original procedure. Genesis-indel is applied to the unmapped reads of 30 breast cancer patients from TCGA. Results show that the unmapped reads are conserved between the two subtypes of breast cancer investigated in this study and might contribute to the divergence between the subtypes. Genesis-indel identifies 72,997 novel high-quality indels previously not found, among which 16,141 have not been annotated in the widely used mutation database. Statistical analysis of these indels shows significant enrichment of indels residing in oncogenes and tumour suppressor genes. Functional annotation further reveals that these indels are strongly correlated with pathways of cancer and can have high to moderate impact on protein functions. Additionally, some of the indels overlap with the genes that do not have any indel mutations called from the originally mapped reads but have been shown to contribute to the tumorigenesis in multiple carcinomas, further emphasizing the importance of rescuing indels hidden in the unmapped reads in cancer and disease studies.

## Introduction

**Figure 1:**
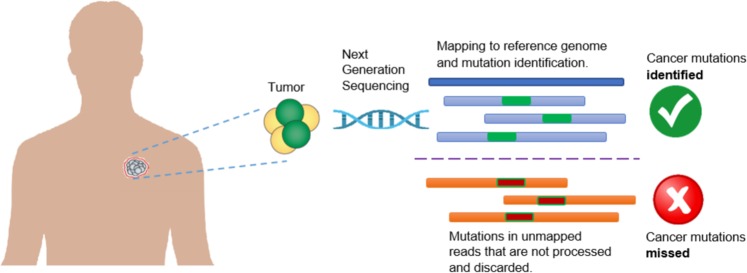
Limitation of current practice in cancer research which discards unmapped reads and therefore misses important mutations containing real biological signal.

Next Generation Sequencing (NGS) facilitates generation of an enormous number of short reads and allows the identification of genomic mutations that cause phenotype changes and genetic diseases such as Mendelian disorders^1^, Acute Myeloid Leukaemia^2^, and Lung cancer^3^. Applications analysing the NGS reads typically start with mapping the short reads against a reference genome and then based on the mapped reads, determine the genetic mutations such as Single Nucleotide Polymorphism (SNP) and sequence variants such as Insertion and Deletion (indel) of bases. Many alignment algorithms have been developed to map the short reads to the reference genome, including MAQ^4^, SOAP^5^, BWA^6^, Bowtie^7^, Bowtie2^8^, SNAP^9^, and SOAP2^10^, to name a few. Although these alignment tools are very efficient in aligning the short reads, a nonnegligible fraction of reads are left unmapped due to (1) structural variants longer than the allowed number of gaps and mismatches by the mapper, (2) sequencing error, or (3) sample contamination^11^. In current practice, these unmapped reads are not used for variant calling and downstream analyses, and thus mutations harboured in these unmapped reads remain hidden from any inference on important phenotype and/or their associations with any disease such as cancer. However, as shown in Fig. 1, some of the “hidden” or “missing” mutations can contain the key for understanding the molecular mechanisms of genetic diseases or cancer and might be used as markers for disease/cancer diagnosis and prognosis.

The consequence of missing the mutations contained in the unmapped reads can lead to inaccurate downstream analyses such as characterizing the tumour evolution in a cancer patient. Some of these missed mutations can be the hallmark of tumours and can be useful for targeted therapy. Therefore, it is critical to identify the mutations in those regions for clinical decision-making as well as for guided personal treatment^12,13^. With this objective in mind, it is essential to inspect the unmapped reads previously excluded from analyses to ensure that none of these essential mutations are missed in those regions of interest.

This paper describes Genesis-indel, a computational pipeline to explore unmapped reads for the systematic identification of indels missed in the original alignments. Note that this pipeline focuses on indels only, the second most abundant form of genetic variation in human populations^14–16^. Despite being a common form of genetic variation in humans, indels have not been studied as thoroughly as SNPs, though they have been identified playing a key role in causing diseases such as Cystic fibrosis^17^, Fragile X Syndrome^18^, acute myeloid leukaemia^2,19,20^, and lung cancer^3^. In addition, insertion of transposable elements such as Alu can affect gene function and change gene expression^21^. Genesis-indel is applied to explore unmapped reads of 30 breast cancer patients from The Cancer Genome Atlas (TCGA)^22^ and identify indels hidden in the unmapped reads of these patient genomes. Results show that unmapped reads can be used to cluster samples to different cancer subtypes. In addition, Genesis-indel can successfully curate the unmapped reads and detect small to large novel high-quality indels that are missed previously and some of these indels are specific to a particular subtype of breast cancer. Functional annotation of the newly identified indels shows that the indels found from unmapped reads are strongly correlated with cancer pathways and may play an important role in cancer progression. Additionally, some of the indels overlap with the genes that do not have any indel mutations called from the originally mapped reads but have been shown to contribute to the tumorigenesis in multiple carcinomas, further emphasizing the importance of rescuing indels from the unmapped reads in cancer and disease studies. Therefore, this study shows great promise in complementing the current procedure of read alignment and variant calling, shedding light on understanding the underlying mechanism of cancer progression and will be useful for clinical decision making.

## Results and Discussion

**Figure 2:**
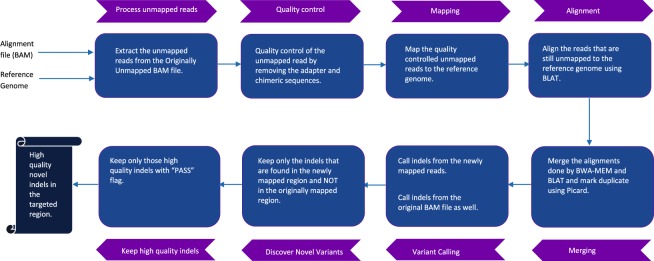
Genesis-indel workflow. The input to Genesis-indel is the alignment file (BAM file) and the reference genome (FASTA format). First, the unmapped reads are extracted from the input BAM and passed to the quality control module. After quality control, the reads are mapped to the reference genome using BWA-MEM. The reads that still remained unmapped are aligned using BLAT. In the merging step, the output of BWA-MEM and BLAT are merged and duplicates are marked using Picard. The merged alignment is then passed to the variant calling module followed by quality filtering of the indels. Finally, the output contains novel high-quality indels rescued from the originally unmapped reads.

Figure 2 shows a schematic representation of the Genesis-indel workflow (see Methods for detail). Genesis-indel is used to identify the novel high-quality indels from the alignment (BAM files) of 30 breast cancer patients deposited in TCGA. These BAM files were originally produced by mapping the raw sequencing reads of these patients to the human reference genome using BWA^6^.

### Existence of a nonnegligible number of originally unmapped reads

**Figure 3:**
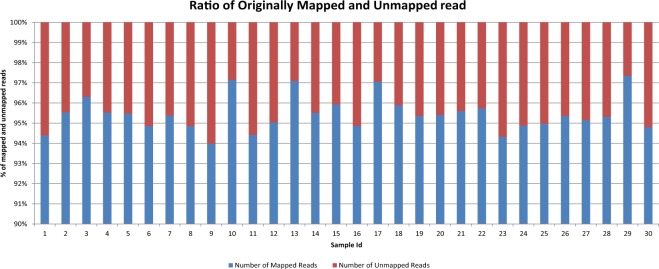
Percentage of mapped and unmapped reads in the original alignment files of the 30 breast cancer patients collected from TCGA.

The alignment file of each patient sample is processed by SAMtools^23^ to extract the “Originally Unmapped” reads. For a given individual investigated here, the number of unmapped reads ranges from 6.6 to 74 million (average = 31.86 million). As shown in Fig. 3, the unmapped reads constitute an average of 5% of the total reads (altogether there are more than 955 million reads unmapped for 30 patient samples) in the original alignment files provided by TCGA. Genesis-indel targets these discarded reads to rescue the indels missed in the original alignment.

### Quality control of the unmapped reads

The extracted unmapped reads are processed for quality control. First, the unmapped reads from all samples are combined and passed to FastQC^24^ to get various statistics of the reads. According to the report produced by FastQC, the originally unmapped reads have some quality issues such as (1) overall poor per base sequence quality, (2) a poor score for per sequence quality, (3) overrepresentation of “N” contents, (4) overrepresentation of Illumina Paired-End PCR Primer 2 due to PCR over-amplification, and (5) other adapter contents (Supplementary Figures 1(a), 2(a), and 3(a)). In most cases, reads that are contaminated with adapter sequences are simply not mapped because of sequencing errors in the adapter sequences. Therefore, removing these contaminated sequences is expected to improve the quality of the unmapped reads. For this reason, Trimmomatic^25^ is applied to the combined unmapped reads from all samples and then FastQC is used again to assess the quality of the reads. As shown in Supplementary Figures 1(b), 2(b), and 3(b), after trimming adapter sequences, many issues were fixed and the quality of the unmapped reads improved significantly. Although there is a low-quality issue with some k-mer noise at the 3′ end of the reads (Supplementary Fig. 4), the mapping is not affected by these k-mers as they are not mapped to the reference genome and hence get discarded during the alignment step. After the quality control by Trimmomatic, for the individuals investigated here, 29.29% to 89.5% of the originally unmapped reads are retained (average = 67.68%) constituting around 647 million reads.

### Mapping the quality controlled unmapped reads

**Figure 4:**
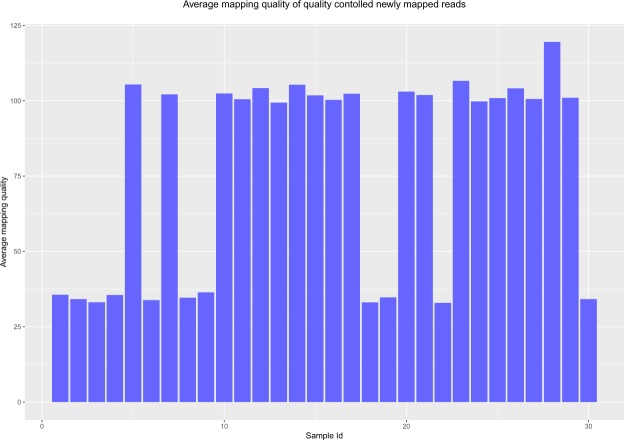
Average mapping quality of the newly mapped reads of all samples.

After quality control, the unmapped reads are mapped to the reference genome using a robust and variant sensitive mapper, BWA-MEM^26^. BWA-MEM can automatically choose between local and end-to-end alignments. It is applicable to map short as well as long reads, and is sensitive in mapping reads with indels. While mapping, unlike other short-read mappers, it allows big gaps potentially caused by structural variants and shows better or comparable performance than several state-of-the-art read mappers to date in terms of speed and accuracy^26^. This mapper is robust to sequencing errors as well. After the reads are aligned by BWA-MEM, some reads still remain unmapped. At this step, another local alignment tool, BLAT (BLAST-Like Alignment Tool)^27^ is used to align these reads. By merging the alignments from BWA-MEM and BLAT, 65.38% of the originally unmapped reads (624,892,089 out of 955,822,913) now get mapped to the reference genome. Out of these newly mapped reads, BWA-MEM mapped 479,064,451 reads and BLAT aligned 145,827,638 reads. As mentioned before, the mapper used by TCGA is BWA, which can map arbitrarily long reads theoretically, however, it has been observed in practice that, the performance in mapping long reads degraded with the increase of the sequencing error rate^28^. By removing the bases with sequencing error and coupling BWA-MEM with BLAT for re-alignment, Genesis-indel manages to map many of the initially unmapped reads. Figure 4 shows the average mapping quality of the newly mapped reads for all samples. For most of the samples, the mapping quality is higher than that of the originally mapped reads (Supplementary Fig. 5).

### Identifying the novel high-quality indels from the newly mapped reads

Genesis-indel uses Platypus^29^ to call indels from the newly mapped reads. Separately, indels are also called from the reads that are originally mapped. Platypus is chosen as it performed the best among other existing indel callers based on real data as reported in a recent review^30^. After variant calling, indels from the newly mapped reads are inspected for any match with the indels found in the originally mapped reads. An indel already in the originally mapped reads can be called again in the newly mapped reads. These “re-identified” indels are discarded to avoid indel redundancy^31^ and the remaining are considered as “novel indels”.

Examination of the flags of the novel indels shows that for many of the indels, Platypus does not produce a high confidence value. Therefore, to consider only the high-quality indels for further analysis, novel indels are filtered again and only those with “PASS” flags are considered for the final result and are termed as “Novel High-Quality indel” (NHQ indel) in this paper. In total, Genesis-indel reports 31,924 NHQ insertions (43.73% of the total NHQ indels) and 41,073 NHQ deletions (56.27% of the total NHQ indels) from the 30 samples investigated here. The deletion to insertion ratio for the NHQ indels is 1.29:1, similar to the deletion to insertion ratio 1.11:1 for the originally mapped reads (7,313,641 insertions and 8,082,055 deletions).

**Figure 5:**
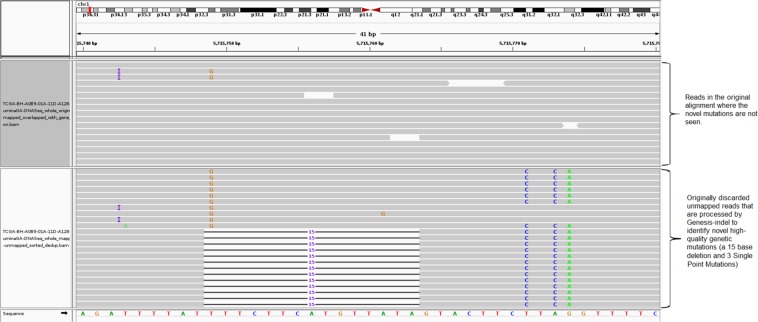
A NHQ indel identified in the newly mapped read but missed in the original alignment. The upper panel shows the originally mapped reads and the lower shows the newly mapped reads.

Figure 5 shows IGV^32^ snapshot of a novel 15-base deletion in Chromosome 1 that is identified in the newly mapped reads (lower panel) but missed in the original alignment (upper panel). Although this paper focuses on indels only, as shown in Fig. 5, new SNPs can also be identified by Genesis-indel in the originally unmapped reads.

### Frameshift indels are more frequent than in-frame types in the NHQ indels

NHQ indels identified here contain 53,623 frameshift and 19,374 in-frame indels, indicating a higher abundance of frameshift indels in the unmapped reads. Frameshift indels are also found more abundant than in-frame indels in the originally mapped reads (13,911,266 vs. 1,481,550). Particularly, frameshift indels of longer length (≥15 bases) are more frequent than in-frame indels of corresponding length (585 insertions, 1,479 deletions vs. 404 insertions, 812 deletions). According to a study by Iengar *et al*.^33^, 75.7% of the COSMIC indels are frameshift indels while only 24.3% are in-frame indels, suggesting that unlike the distribution of coding indels in the genome of healthy people, frameshift indels dominate in cancer genomes. Because frameshift mutations are common in cancer patients and may increase the susceptibility to cancers and other diseases by causing loss of significant fractions of proteins^33–36^, the NHQ frameshift indels newly uncovered by Genesis-indel may harbour important signals for linking indels to cancer or diseases and provide researchers new insights into the underlying mechanisms.

### NHQ indels have significantly different length distribution than indels in the originally mapped reads

**Figure 6:**
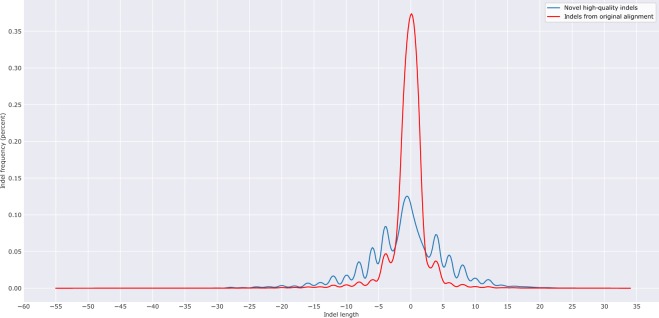
Length Distribution of the NHQ indels and indels from the originally mapped reads for all samples. Here a negative value indicates the deletion length.

Figure 6 shows the distribution of the length of the NHQ indels analysed here. It is observed that both insertion and deletion frequencies decrease with the increase of indel size. The longest NHQ insertion and deletion are 28 and 45 bases, respectively (34 and 55 bases for the indels from the originally mapped reads). It is expected that the novel indels would be long as they might have been missed because the lengths might exceed the number of gaps and mismatches allowed by the mapper. Surprisingly, as shown in Fig. 6, most of the newly discovered indels (91% of insertion and 88.1% of deletion) are short (≤10 bases), indicating the limitation of the mapper used in the TCGA project. This figure also shows that NHQ indels have higher relative frequency than indels from the originally mapped reads for both insertion (3 to 28 base) and deletion (3 to 45 base). Nonetheless, a Pearson’s chi-square test (i.e., testing for homogeneity in contingency table) shows that the length distribution of the NHQ indels is significantly different than that of the indels identified from the originally mapped reads (p-value < 2.2e-16).

### Newly mapped reads can add more support to indels not recognized in the originally mapped reads

**Figure 7:**
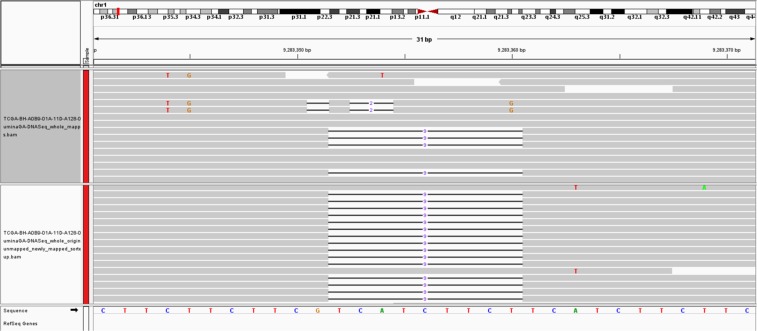
An example of a 9-base deletion which is not initially called from the original alignment due to lack of read-support but later called after the mapping of originally unmapped reads. Here the upper panel shows the original alignment and lower panel shows the alignment of the newly mapped reads.

Most variant calling programs rely on hard evidence for indels marked in the alignment and therefore require a minimum number of reads to support an indel. This step is required to distinguish real variants from the artefacts of sequencing errors. As shown in Fig. 7 (upper panel), a 9-base deletion cannot be called from the original alignment due to lack of read support. After mapping the quality controlled originally unmapped reads through the Genesis-indel pipeline, such indels get enough read support and hence are called by the variant caller (lower panel). This provides an example scenario for how these indels are missed initially but got rescued by leveraging the unmapped reads.

### Clustering of the samples based on quality-controlled unmapped reads

**Figure 8:**
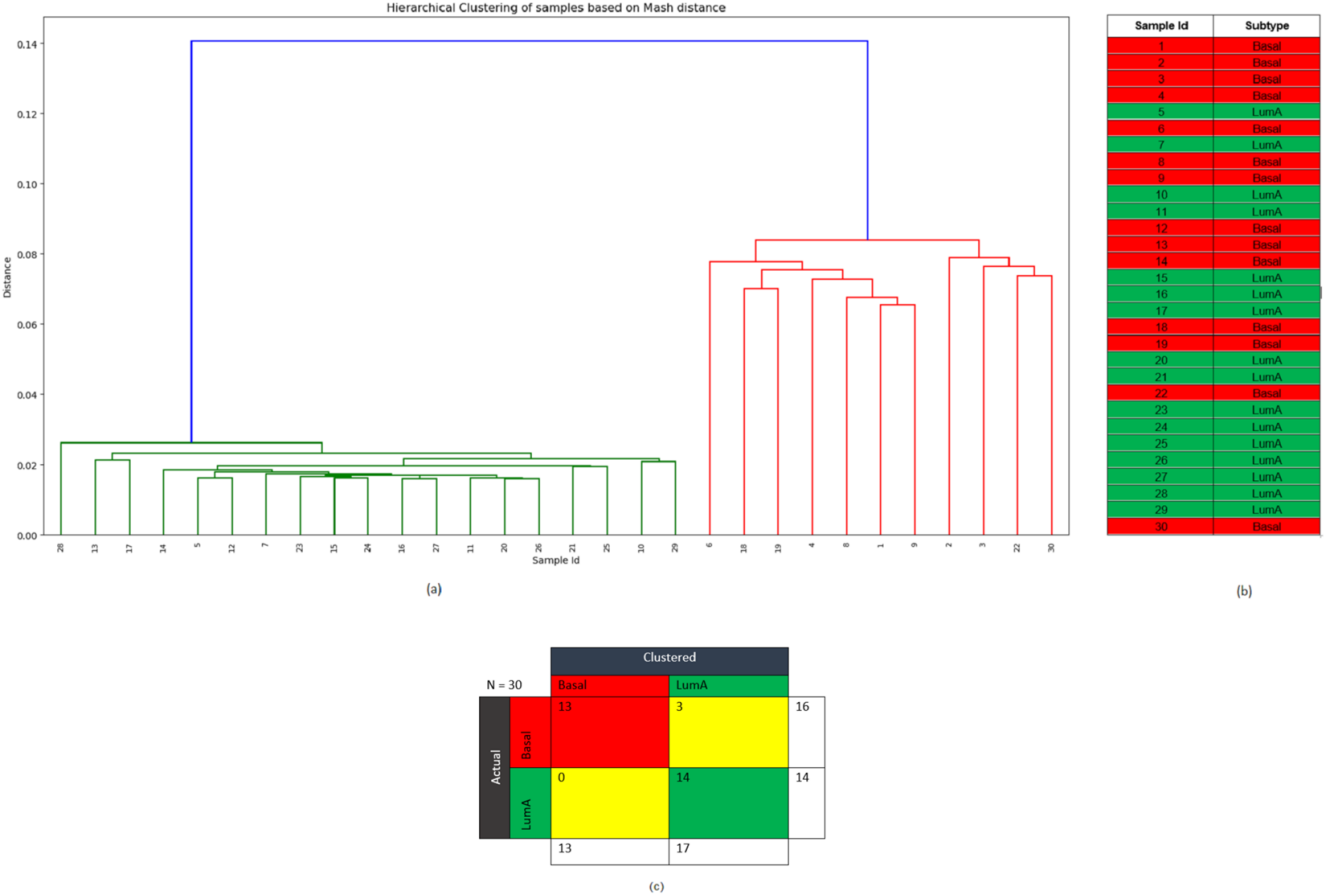
(**a**) Hierarchical clustering of the samples based on the pairwise Mash distance of the unmapped reads from each sample. (**b**) PAM50 subtype of the samples from TCGA. Here, the red colour corresponds to Basal and green colour corresponds to LumA subtype. (**c**) The confusion matrix.

The samples are compared pairwise using the quality controlled unmapped reads in order to identify biologically relevant signals and to cluster the samples based on the number of similar reads. Pairwise distance is calculated for the unmapped reads from each sample using Mash, a distance estimator based on MinHash^37^. This pairwise distance is then used to cluster the samples. Figure 8 shows the hierarchical clustering of the samples based on Mash distance. The clustering results are then compared with the samples’ PAM50 subtypes collected from TCGA^38^. Out of the total 30 samples, 16 belong to the Basal subtype and the remaining 14 belong to LumA. As shown in Fig. 8, all but three samples (samples 12, 13, and 14) cluster with the samples of their respective subtype. These three samples belong to Basal subtype but clustered with the samples from LumA subtype. The result reveals that the unmapped reads are most commonly shared among the samples of the same subtype and suggests that these unmapped reads might contribute to the divergence between the two subtypes investigated here. This result also implies that perhaps there is a subtype-specific common cause of mapping failure. These results show that the sets of unmapped reads contain sequence information specific to sample subtype and hence leveraging such information may help understand or interpret the related biological questions.

### Subtype-specific indels from the NHQ indels

All except three samples (samples 12, 13, and 14) of the Basal subtype contain 5,818 indels on average and LumA samples contain 473 indels on average. Samples 12, 13, and 14 contain 484, 415, and 535 indels, respectively, i.e., similar to the number of indels found in the LumA samples. Similar phenomena are also observed for these three samples in the indels from the originally mapped reads, giving more evidence that these samples actually belong to LumA subtype but were mislabelled as Basal, consistent with the result of clustering based on unmapped reads. In addition, the number of newly mapped reads in Samples 12, 13, and 14 are 3.6, 2.5, and 3.5 million, respectively, which is closer to the number of newly mapped reads in LumA samples (average number of newly mapped reads = 3.3 million) than to the Basal samples (average number of newly mapped reads = 37.89 million). This suggests possible subtype mislabelling of these samples.

NHQ indels are checked to see if they are specific to any of the two subtypes (Basal and LumA) investigated here. An indel is defined as specific to a subtype when it is found in the samples of one subtype and not in the samples of the other subtype. Among the 72,997 NHQ indels, 89 are found to be Basal specific indels and none is found to be LumA specific.

### NHQ indels overlapped with the oncogenes and tumour suppressor genes

To see which oncogenes and tumour suppressor genes are frequently affected by the newly discovered indels, a list consisting of 142 protein-coding genes (79 oncogenes and 63 tumour suppressor genes, see Methods) is overlapped with the NHQ indels using BEDtools^39^. In total, 62 out of these 142 genes overlapped with these indels. Among these 62 genes, 32 are oncogenes and the remaining ones are tumour suppressor genes. Table 1 lists the top ten genes with the highest number of indels identified in them. RUNX1 (Runt Related Transcription Factor 1), a protein-coding tumour suppressor gene, has the highest number of indels (54) and thus likely contains important signature of breast cancer. RUNX1 has received attention as a gene fusion in acute myeloid leukaemia (AML)^40,41^. Although a putative link to breast cancer has recently emerged^42^, RUNX1 has not gained enough attention and its role in breast cancer still remains elusive^43^. One reason for the understudy of the RUNX1 gene is the underpowered expression profile studies as identified by Janes *et al*.^44^. Another reason, as the result shows here, could be because of not discovering the indels hidden in the unmapped reads. This study provides new evidences to re-examine the role of RUNX1 in breast cancer, as a complement to the study performed by Janes *et al*.^44^.

**Table 1:** Top ten oncogene and tumour suppressor genes and the number of indels identified in these genes.

Gene	Number of Indel
RUNX1	54
SYK	13
CBLB	11
ETV4	11
CCND3	10
ETV6	9
MAML2	7
PIK3CA	7
BMPR1A	6
EGFR	5

Frameshift indels are more abundant than in-frame indels in both oncogenes and tumour suppressor genes. Some genes contain only in-frame indel and some contain only frameshift indels. As shown in Table 2, out of the 62 genes overlapped with the NHQ indels, 46 contain either in-frame or frameshift indels. The remaining 16 genes contain both in-frame and frameshift indels. As shown in the previous section, frameshift indels are the dominant type of indels and RUNX1 contains the maximum number of indels for both in-frame (12) and frameshift (42) among all genes investigated here, making it an important candidate for breast cancer marker.

**Table 2:** List of oncogenes and tumour suppressor genes containing either in-frame or frameshift NHQ indels.

Indel Type	Gene
In-frame	Oncogenes (5): HMGA2, TPR, RAF1, ROS1, FGFR2
	Tumour suppressor genes (3): EXT1, CREB1, GPC3
Frameshift	Oncogenes (19): ATF1, CBLB, AKT2, LMO2, TET2, ETV4, BCR, MAF, SMO, PPARG, CARD11, DDX6, PLAG1, EGFR, ABL1, NTRK1, BCL11A, BCL2, FGFR1
	Tumour suppressor genes (19): RB1, SMARCB1, FLT3, BRCA1, CDH1, SUFU, CHEK2, ARHGEF12, FBXW7, MSH2, NUP98, SUZ12, NPM1, BCL11B, IDH1, EXT2, NR4A3, ATM, BMPR1A

### NHQ indels mostly alter cancer-related genes than noncancer-related genes

A list of whole genome protein-coding genes not containing the oncogene and tumour suppressor gene is generated from the whole genome gene list produced by GENCODE (version 28 lift37). This list contains 20,172 genes and among these, 6,829 genes are found overlapped with the NHQ indels. A hypothesis testing is done to compare the proportion of NHQ indels appearing in cancer-related genes (oncogene and tumour suppressor gene) to that in noncancer genes. Statistically, the hypothesis being tested is as follows,

$${H}_{0}:\,{p}_{1}\le {p}_{2}$$

$${H}_{1}:\,{p}_{1} > {p}_{2}$$

where *P*_1_ denotes the proportion of oncogenes and tumor suppressor genes that overlapped with the NHQ indels and *P*_2_ denotes the proportion of noncancer genes that overlapped with the NHQ indels.

Given the data observed (62 out of the total 142 oncogenes and tumour suppressor genes are overlapped with the NHQ indels, and 6,829 out of the total 20,172 noncancer genes are overlapped with the NHQ indels), a z-test for testing the difference in two proportions reports an observed z value of 2.35, yielding a p-value of 0.0094. Equivalently, a chi-square test of homogeneity based on the 2 × 2 contingency table gives a p-value of 0.0177 with Yates’ continuity correction. Both results show that the null hypothesis, *H*_0_:*P*_1_ ≤ *P*_2_ can be rejected at nominal level α = 0.05. Therefore, the proportion of cancer genes overlapping with NHQ indels is significantly higher than that for noncancer genes.

An alternative approach by permutation test is also done to see if the NHQ indels have a higher enrichment in cancer genes (oncogenes and tumour suppressor genes) than in noncancer genes. For this test, from the list containing 20,172 genes (the whole genome gene list not containing the oncogene and tumour suppressor genes), 142 genes (number of total oncogene and tumour suppressor genes) are sampled randomly and checked for the number of genes that overlap with the NHQ indels. Repeating the sampling 1,000 times yielding a distribution for the number of genes overlapping with the NHQ indels. Out of the 1,000 sets, the number of overlaps higher than 62 (the observed number of overlaps) only occurs 9 times and therefore, the permutation test p-value is 9/1000 = 0.009 (which is also consistent with the p-value from the z-test above). Again, the null hypothesis can be rejected, and therefore, it can be concluded that there is a significant enrichment of the NHQ indels in cancer genes, further suggesting that the NHQ indels may harbour important genetic mechanisms for breast cancer.

### Annotating the NHQ indels using Variant Effect Predictor (VEP)

For this analysis, the NHQ indels are annotated using Variant Effect Predictor (VEP)^45^. 16,141 of the indels are identified as novel, i.e., not annotated in the Ensembl variation database consisting of dbSNP, Cancer Gene Census, ClinVar, COSMIC, dbGap, DGVa etc. This indicates the significance of this study in rescuing these indels from the discarded reads that can potentially be annotated.

**Figure 9:**
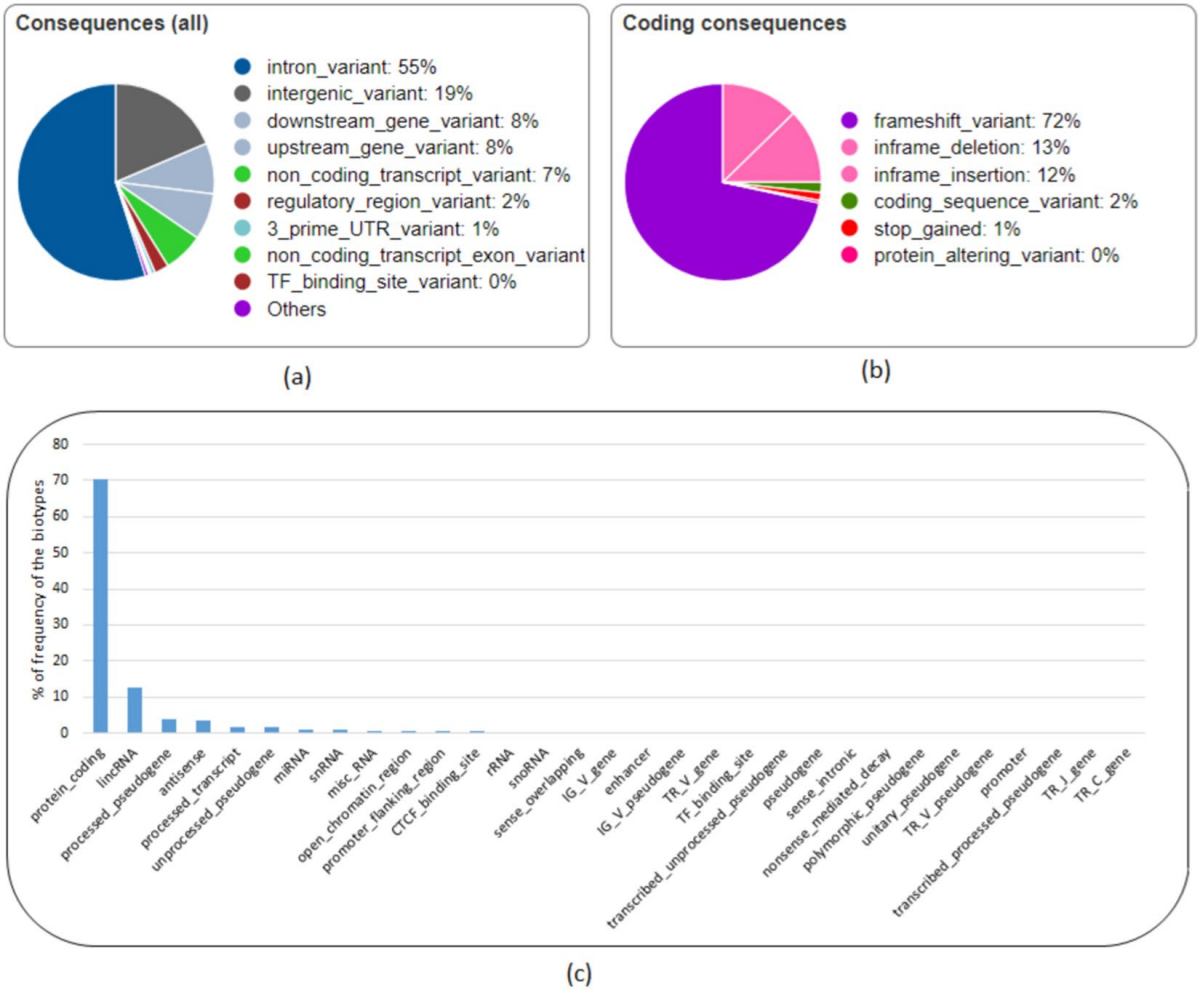
Analysis of the NHQ indels using Variant Effect Predictor (VEP). (**a**) All consequences, (**b**) coding consequences, (**c**) distribution of biotype of the features overlapped with the NHQ indels.

The NHQ indels overlapped with 15,229 genes, 32,335 transcripts, and 2,136 regulatory features. As shown in Fig. 9(a), around 75% of the NHQ indels are in the non-coding regions located in the intron or intergenic region. Figure 9(b) shows that 72% of the indels in the coding regions are frameshift indels that cause a disruption of the translational reading frame and can have a disruptive impact in the protein by causing protein truncation and/or loss of function. In addition, a small amount of the indels are “Splice donor variants” changing the 2-base region at the 5’ end of an intron and can have a similar impact as frameshift indels. 25% of the indels are in-frame indels having a “moderate” impact in the protein by not disrupting the protein but changing the effectiveness of that protein. 70% of the NHQ indels having disruptive and moderate impact overlap with the protein-coding transcripts, the leading biotype of all features (Transcripts, Regulatory Features, and Motif Features) as shown in Fig. 9(c). Out of the remaining indels, 37.44% (12.48% of the total NHQ indels) have a “modifier” impact that overlap with long intergenic RNA transcripts (lincRNA). LincRNAs are noncoding transcripts with a length longer than 200 nucleotides and are the largest class of noncoding RNA molecules in the human genome. There is an emerging evidence that noncoding RNAs regulate gene expression by influencing chromatin modification, mRNA splicing, and protein translation^46,47^ as well as contribute to mammary tumour development^48,49^ and progression. Therefore, the NHQ indels overlapped with these transcripts deserve more attention and studying these indels has biological significance.

### Functional annotation of the genes overlapping with the NHQ indels

Functional annotation of the genes overlapping with the NHQ indels using David^50^ (version 6.7) shows strong correlation with “Pathways in Cancer” (Fisher Exact p-value: 6.2 × 10^−5^), “PI3K-Akt signalling pathway” (Fisher Exact p-value: 1.5 × 10^−4^), RAP1 signalling pathway (Fisher Exact p-value: 10^−4^), and RAS signalling pathway (Fisher Exact p-value: 4.7 × 10^−3^). A previous study shows that components of the PI3K-Akt signalling pathway are recurrently altered in cancers and the survival signals induced by several receptors are mediated mainly by this pathway^51^. Ras-associated protein-1 (RAP1) is an important regulator of cell functions and has been found playing a vital role in cell invasion and metastasis in cancers^52^. The signalling pathways involving RAS protein can contribute to tumour growth, survival, and spread, and play a crucial role in the pathogenesis of other hematologic malignancies as well^53,54^. Therefore, these results suggest that the newly found indels interacting with these genes may participate in cancer-related biological processes and play an important role in cancer progression.

### Genes missed in the original mapping but found in NHQ indels show association with cancer and other diseases

There are 42 genes overlapping with the NHQ indels but not with the indels from the originally mapped reads. Table 3 lists the genes with their types. Functional annotation of the protein-coding genes shows that these genes are related to biological process such as immune response, protein localization, protein transport, regulation of transcription, and regulation of RNA metabolic process which can control molecular functions such as antigen binding, peptide binding, MHC protein binding, and peptide-antigen binding. In addition, these genes are associated with protein domain such as Immunoglobulin subtype and Krueppel-Associated Box (KRAB)–Zinc Finger Protein (ZFP). Immunoglobulin subtype is involved in cell-cell recognition, cell-surface receptors, muscle structure, and the immune system^55^ and therapy targeting this protein domain has been used for liver cancer^56^, breast cancer^57^, and Follicular Lymphoma^58,59^. Krueppel-Associated Box (KRAB)–Zinc Finger Protein (ZFP) is the largest class of transcription factors in the human genome^60^ and is largely involved in tumorigenesis^61^.

**Table 3:** Name and type of the genes that overlap with the NHQ indels but not with the indels from the originally mapped reads.

Gene Type	Gene Name
Antisense	RP11-534L20.5, JMJD1C-AS1, CTB-22K21.2, RP11-1079K10.4, RP11-16N11.2, RP11-26P13.2, RP11-1299A16.3, RP1-16A9.1.
LincRNA	RP5-1065P14.2, RP11-309G3.3, RP11-382D12.1, RP11-14C22.3, RP11-386I8.6, LINC00379, RP3-503A6.2, RP3-416J7.4, RP11-100L22.4.
miRNA	MIR4477A.
Pseudogene	RP5-857K21.7, RP11-428G5.7, BNIP3P1, RP11-713H12.2, VN1R90P, MLLT10P1, UNC93B3, TBCAP3, CTD-2158P22.1, RP11-823P9.3, RP3-416J7.1, CYP4F44P, OR5BH1P, PABPC1P3, CASKP1, TRBV12-1, TRBV12-2.
Protein coding	OR10G8, ZNF26, ZNF84, KIF20A.
snRNA	RNU1-59P, RNU6-377P, RNU1-36P.

A PubMed search returned results for three genes namely KIF20A, BNIP3P1, and ZNF84.

**Figure 10:**
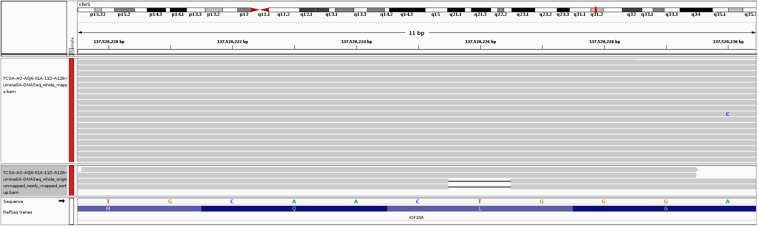
A 1-base deletion in the exon of KIF20A gene which is not initially called from the original alignment but called after the mapping of originally unmapped reads. The upper panel shows the original alignment and lower panel shows the alignment of the newly mapped reads.

Kinesin family member 20A (KIF20A), also known as RAB6KIFL, is a member of the kinesin superfamily of motor proteins, a conserved motor domain which binds to microtubules to generate the energy required for trafficking of proteins and organelles during the growth of numerous cancers^62,63^. KIF20A is found overexpressed at both the mRNA and protein levels than the normal counterparts in breast cancer^64–66^ and also in several other cancers including gastric cancer^67^, bladder cancer^68,69^, pancreatic cancer^70–72^, hepatocellular cancer^73^, lung cancer^74^, glioma^75^, and melanoma^76^. The overexpression of KIF20A is significantly associated with poor survival of breast cancer patient^64,65^ and drug resistance^65,77^. Similar phenomena are observed with other cancer patients as well^67,69,70,72,74,78^. Silencing or knockdown of KIF20A can significantly inhibit cell proliferation and cancer progression^71,79^. Therefore, KIF20A has been suggested as a direct therapeutic target^71,80^, and KIF20A-derived peptide has been used in immunotherapy in clinical trials to improve the prognosis of cancer patients^62,75,81–84^. Although KIF20A has a strong association with breast cancer, no mutation is found in this gene from the originally mapped reads which shows the limitation of the current approach. This limitation, however, can be alleviated by exploring the unmapped reads. Besides cancer, KIF20A is found associated with heart disease in infants. A recent study by Louw *et al*.^85^ identified an undescribed type of lethal congenital restrictive cardiomyopathy, a disease affecting the right ventricle of two siblings. Exome sequencing analysis of these affected siblings and their unaffected sibling revealed two compound heterozygous variants in KIF20A; a maternal missense variant (c.544 C > T: p. R182W) changing an arginine to a tryptophan and a paternal frameshift deletion (c.1905delT: p. S635Tfs15, in exon 15) that introduces a premature stop codon 15 amino acids downstream. Louw *et al*.^85^ validated the variants by Sanger sequencing, found the presence of both variants in the affected siblings, and confirmed a heterozygous carrier status in both parents. In addition, both variants were absent in the unaffected sibling. The C > T missense SNP does not let KIF20A support efficient transport of Aurora B as part of the chromosomal passenger complex causing Aurora B trapped on chromatin during the cell division and hence it fails to translocate to the spindle midzone during cytokinesis. This claim is verified by Louw *et al*.^85^ in the zebrafish model where translational blocking of KIF20A resulted in a cardiomyopathy phenotype. A similar congenital restrictive cardiomyopathy is also identified to be caused by the deletion resulting in loss-of-function of KIF20A^85^. Despite such significance, these two variants that affect protein function were absent in the population control exome such as ExAC Browser database, a catalogue of genetic data of 60,706 humans of various ethnicities^86^. The missense variant was found in two individuals from South Asia and Europe and the frameshift deletion was present in 32 individuals of African descent^85,87^. This observation supports the claim that clinically important mutations can be missed and one of the reasons might be because of overlooking the unmapped reads. By exploring the unmapped read of 30 breast cancer patients, Genesis-indel finds a frameshift deletion of T (chr5:137520225 CT -> C) that overlaps with the exon of KIF20A gene (Fig. 10). Note that, in Fig. 10, only two out of four reads support the deletion where as in Fig. 7, a 9 base deletion is not called from the originally mapped reads although 4 reads support that deletion. The reason is, the single base deletion is supported by 50% of the reads aligned in that region whereas the 9-base deletion in the originally mapped reads (Fig. 7 upper panel) is supported by 25% of the aligned reads which is possibly lower than the default threshold set by the variant caller.

Among the remaining genes, BCL2 interacting protein 3 pseudogene 1 (BNIP3P1) is found to be upregulated in patients with breast cancer Brain Metastases when compared to breast cancer (76% vs. 24%) or compared to Primary Brain Tumours (74% vs. 26%)^88^ and is suggested to be used as a molecular biomarker for breast cancer Brain Metastases. Zinc Finger Protein 84 (ZNF84) is found significantly associated with tumour size and TNM (Tumour, Node, Metastases) staging for cervical cancer and squamous cell carcinoma and *in vitro* validation shows that it promotes cell proliferation via AKT signalling pathway^89^. Although the literature does not show any association between these genes and breast cancer, it is worth exploring due to their association with other cancers.

Out of the 42 genes, two LincRNAs namely RP3-416J7.4 and RP11-386I8.6 contain the same number of indels as the protein-coding genes. Although little is known about their association with breast cancer, analysis using TANRIC^90^ on TCGA-BRCA data reveals that these two LincRNAs are differentially expressed (t-test p-value = 0.000023337 and 0.003812, respectively) between the carriers and non-carriers of somatic mutations in the TP53 gene, a tumour suppressor gene spontaneously found altered in breast carcinomas^91^.

While this paper shows the significance of uncovering NHQ indels from the originally unmapped reads in patients with breast cancer, there are few limitations. Firstly, this study is conducted by using a computational pipeline. Though the pipeline is computationally feasible and results are convincing as well as supported by experimentally validated literature, it lacks some validation experiments. Integrated Genome Viewer (IGV) clearly shows the novel high-quality indels discovered by Genesis-indel. Use of IGV is a well-accepted approach for computational validation of variants like SNPs, indels, and SVs. Nonetheless, *in vivo* validation is essential to govern the clinical importance of the newly identified indels. Secondly, filtering indels solely based on the “PASS” flag may cause missing rare variants. Therefore, an algorithm such as ForestQC^92^ that combines traditional variant filtering approach with machine learning algorithm to determine the quality of the variant can be incorporated to the present pipeline to improve the quality control procedure and achieve better results. Thirdly, if the reads are initially quality controlled and mapped with BWA-MEM, in that case, Genesis-indel will not have many unmapped reads to analyse and will produce results solely based on the few reads aligned by BLAT.

## Conclusion

This paper emphasizes the interest of studying unmapped reads to cope with potential loss of important information and describes Genesis-indel, a computational pipeline to rescue novel high-quality indels by exploring unmapped reads that are normally discarded from the downstream analysis.

Analysing the whole genome DNA alignment of 30 breast cancer patients from TCGA reveals a nonnegligible number of unmapped reads that are overlooked earlier. After mapping the unmapped reads to the reference genome, Genesis-indel finds 72,997 novel high-quality indels of diverse lengths and 16,141 have not been annotated in any of the genetic variation database used by Ensembl. These novel high-quality indels are mainly enriched in frameshift indels and have high to moderate impact in the protein. These indels mostly alter the oncogenes and tumour suppressor genes and overlap with genes significantly related to different cancer pathways. Moreover, these indels overlap with genes not found in the indels from the originally mapped reads and functional annotation shows that these genes contribute to the development and growth of tumour in multiple carcinomas. Therefore, these findings collectively suggest that complete characterization of these indels is essential for downstream cancer research. Genesis-indel is expected to be highly useful for uncovering the missed indels that can be further explored for clinical decision making.

## Methods

### The Genesis-indel pipeline

Genesis-indel is designed to leverage unmapped reads from an alignment with the goal to rescue indels that are hidden in the discarded unmapped reads. Figure 2 shows a schematic representation of the Genesis-indel workflow. The input to Genesis-indel is the alignment file (BAM file) of the patient genome and the reference genome. In the pre-processing step, Genesis-indel extracts the unmapped alignment by checking the alignment flag using SAMtools (version 1.4)^23^. From this, it extracts the “Originally Unmapped” reads using SAMtools and stores the reads in a FASTQ file. This FASTQ file is then processed by Trimmomatic (version 0.36)^25^ to do the quality control of the unmapped reads by removing adapter sequences. In this experiment, the Illumina adapter, TruSeq2 for single-end reads are removed. Moreover, low quality or N bases where the base quality is below 3 are removed from both ends of the reads (LEADING:3, TRAILING:3). Reads are scanned with a 4-base wide sliding window and are cut when the average quality per base drops below 15 (SLIDINGWINDOW: 4:15). Reads with length below 36 bases are dropped (MINLEN:36). These quality controlled single-end reads are used as the input to the mapper in the next step.

The quality controlled unmapped reads are mapped to the reference genome using BWA-MEM (version 0.7.15-r1140)^26^, a sensitive mapper to map reads with indels. After the reads are aligned by BWA-MEM, some reads still remain unmapped. These reads are aligned to the reference genome using BLAT (BLAST-Like Alignment Tool)^27^, another sensitive local alignment tool. At the end of this step, the alignments from BWA-MEM and BLAT are merged. The resultant alignment is sorted and indexed using SAMtools and duplicates are marked in the newly mapped reads using MarkDuplicates tool from Picard (version 1.65)^93^. After read alignment and marking duplicates, indels are called using Platypus (version 0.7.9.1). Separately, indels are also called from the original (input) BAM file. Indels found only in the newly mapped reads and not in the original alignment are reported as novel indels. After identifying the novel indels, another step of filtering is done to keep only the high-quality indels, i.e., the indels that are called with high confidence by Platypus. Therefore, only the indels with the “PASS” flags are reported at the final step. These are the Novel High-Quality indel (NHQ indels) reported in the final output and selected for downstream analysis.

### Preparing a list of oncogene and tumour suppressor genes

A list of oncogenes and tumour suppressor genes is obtained from an online resource^94^, a list compiled from the CancerGenes^95^. While preparing the list, if a gene is marked as both an oncogene and a tumour suppressor gene in CancerGenes, a literature search is performed to determine the gene’s role in tumour development. Any gene with an ambiguous role as an oncogene or tumour suppressor gene is excluded from the list. The final list contains 79 oncogenes and 63 tumour suppressor genes. The start and end positions of the genes are obtained from GENCODE (version 28 lift37). Supplementary Table S1 contains the list of the genes and their positions.

### Software availability and system requirements

Genesis-indel is implemented in C++ and can run on any operating systems that have a C++ compiler. The source code and the command line version of Genesis-indel are freely available at https://github.com/mshabbirhasan/Genesis-indel. Users are welcome to report bugs and provide comments through the issue tracker on GitHub. The README describes the command line options available in Genesis-indel with examples. Although Genesis-indel uses BWA-MEM as the mapper and Platypus as the default variant caller, future version will allow the user flexibility to customize the program and use the mapper and caller of their choice by making small modifications to the script.

## Supplementary information

Supplementary Table S1

Supplementary Table S2

Supplementary Materials

## Data Availability

No new data sample is generated for this study. The alignment file (BAM) for the 30 breast cancer patients are obtained from The Cancer Genome Atlas (TCGA) project (https://portal.gdc.cancer.gov/). Supplementary Table S2 lists the TCGA Sample Barcode and alignment filename for the patients. The reference genome used is Homo_sapiens_assembly19.fasta, the same reference used by TCGA to align the reads. The annotation of the genes is collected from GENCODE (version 28 lift37). All other data supporting the findings of this study are available in this article and in the supplementary materials. These data are also available from the authors upon request.
