# Supplementary material for: Uncovering missed indels by leveraging unmapped reads
Source: Sci Rep. 2019 Jul 31;9:11093. doi: 10.1038/s41598-019-47405-z (PMC6668410; doi:10.1038/s41598-019-47405-z)
Supplement: Supplementary file 3 — Supplementary Materials [file 41598_2019_47405_MOESM3_ESM.pdf]

Supplementary Materials

Uncovering missed indels by leveraging unmapped reads

Mohammad Shabbir Hasan[1], Xiaowei Wu[2], and Liqing Zhang[1*]

[1]Department of Computer Science

[2]Department of Statistics

Virginia Tech, Blacksburg, VA 24061.

Email: shabbir5@vt.edu, xwwu@vt.edu, lqzhang@vt.edu

[*] Corresponding Author

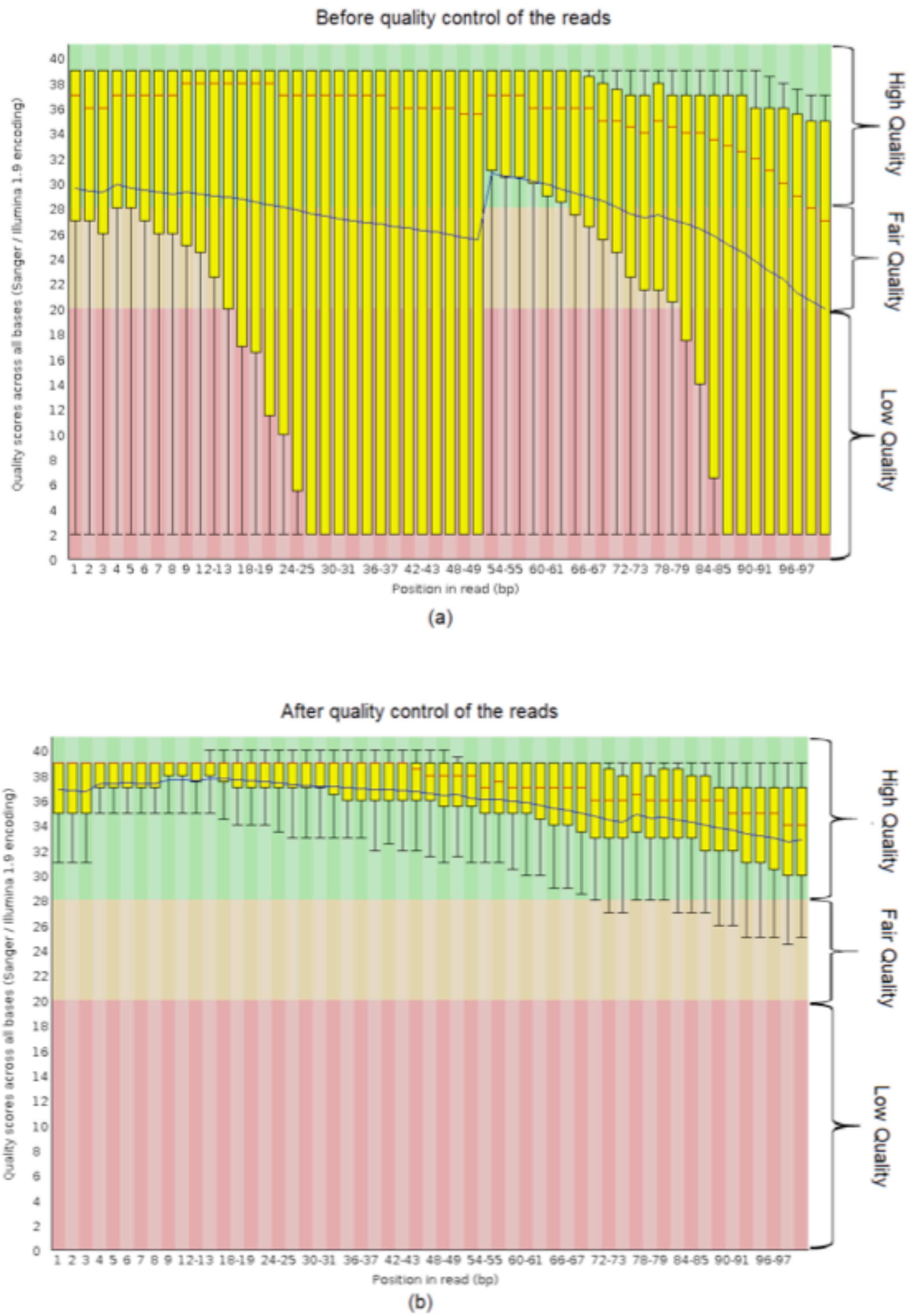

Supplementary Figure 1: Per base sequence quality (a) before and (b) after the quality control of the reads.

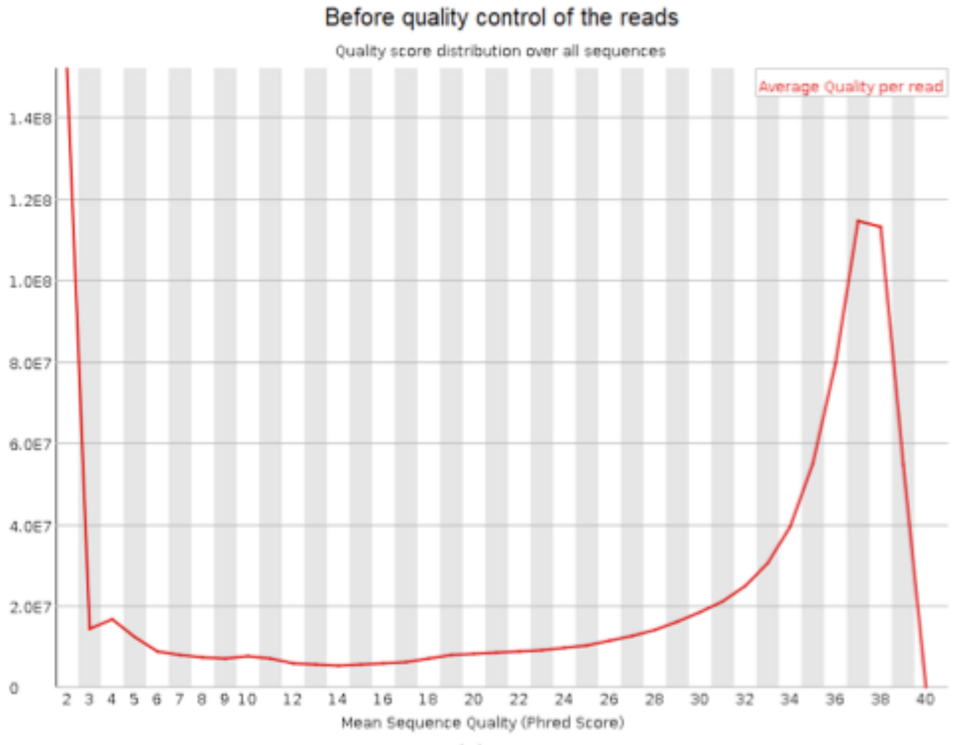

(a)

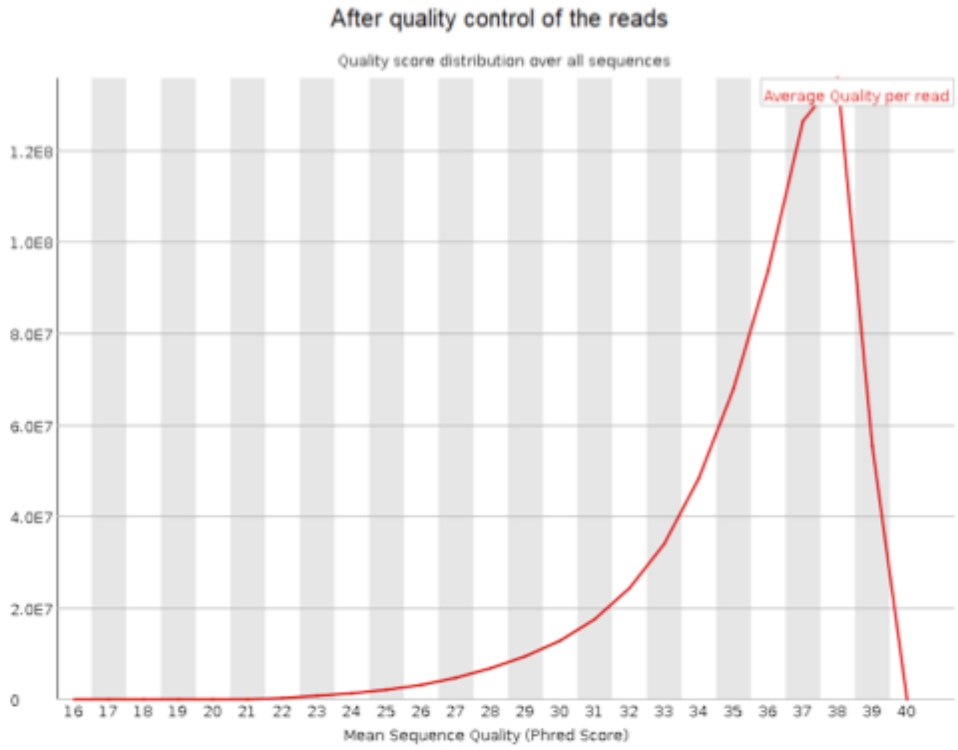

(b)

Supplementary Figure 2: Per sequence quality score (a) before and (b) after the quality control of the reads.

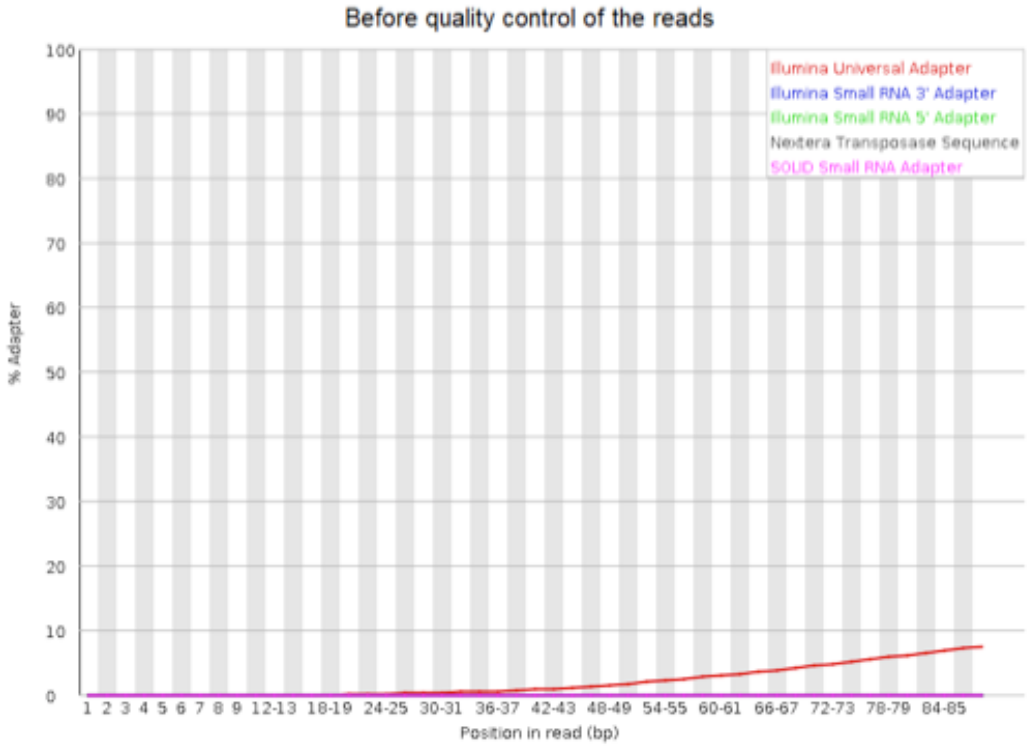

(a)

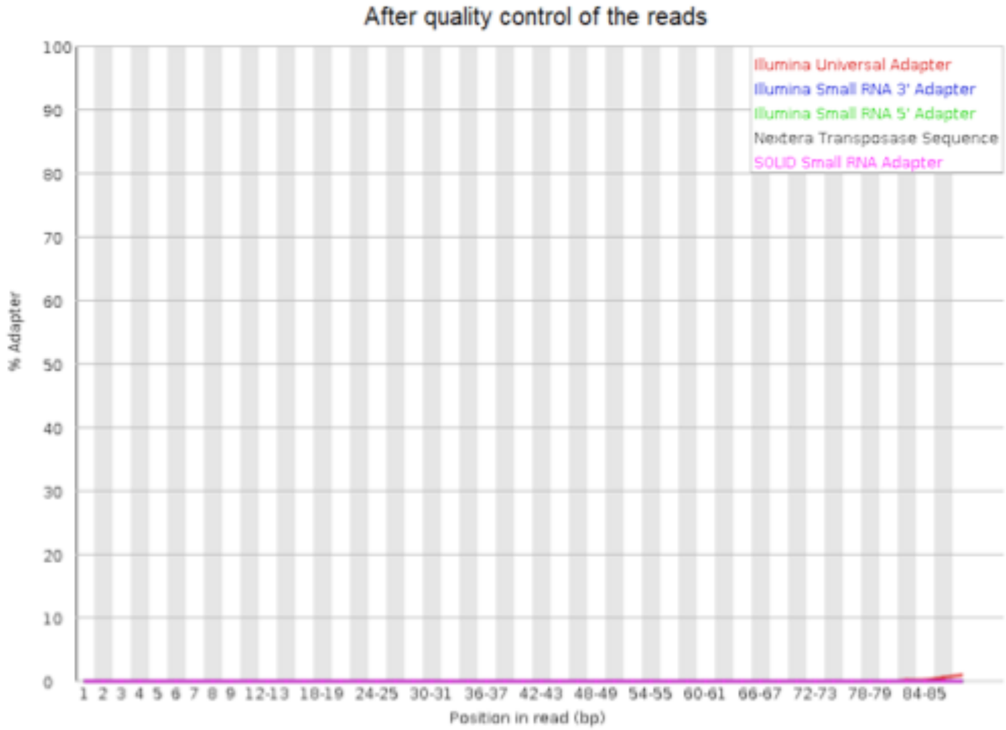

(b)

Supplementary Figure 3: Percentage of Adapter contents in the reads (a) before and (b) after the quality control.

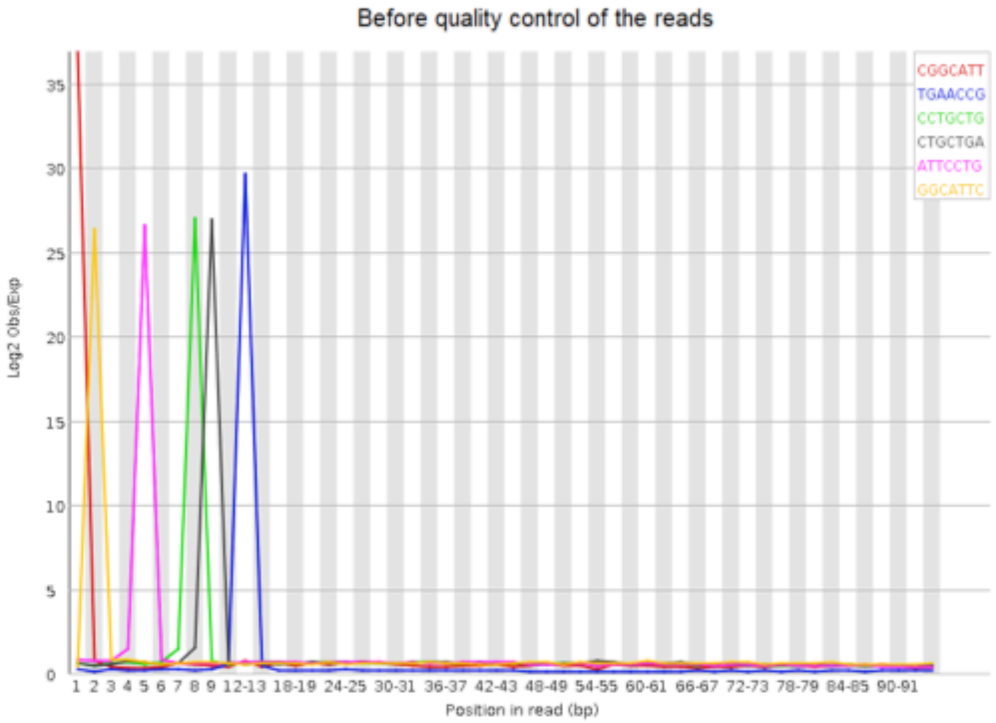

(a)

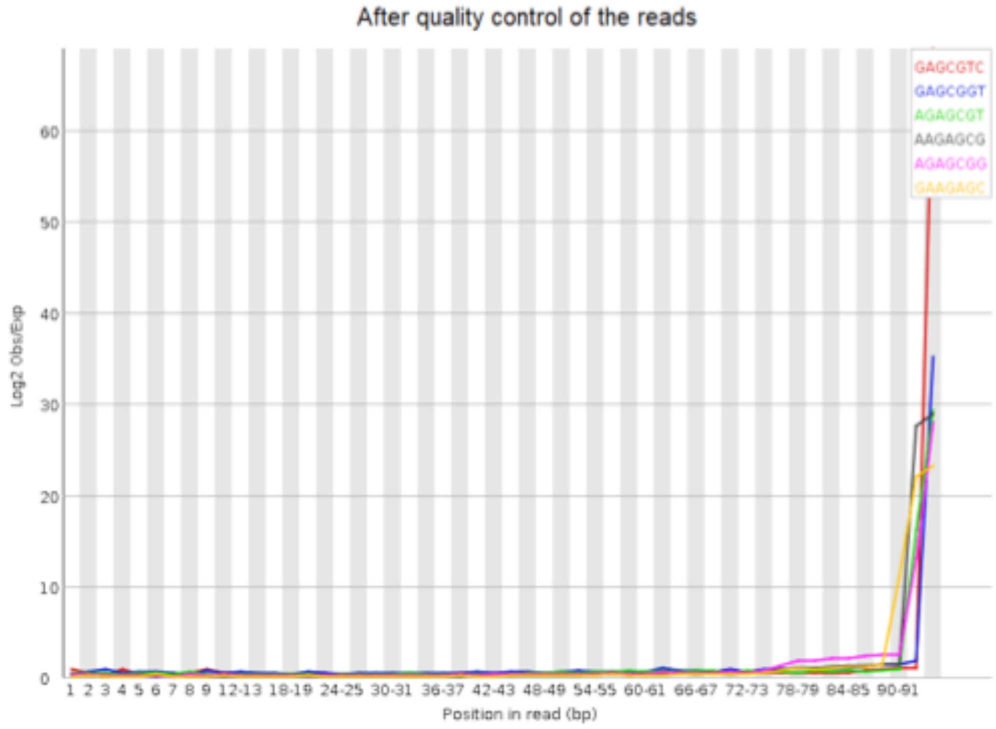

(b)

Supplementary Figure 4: Observance of different k-mers in the reads (a) before and (b) after the quality control.

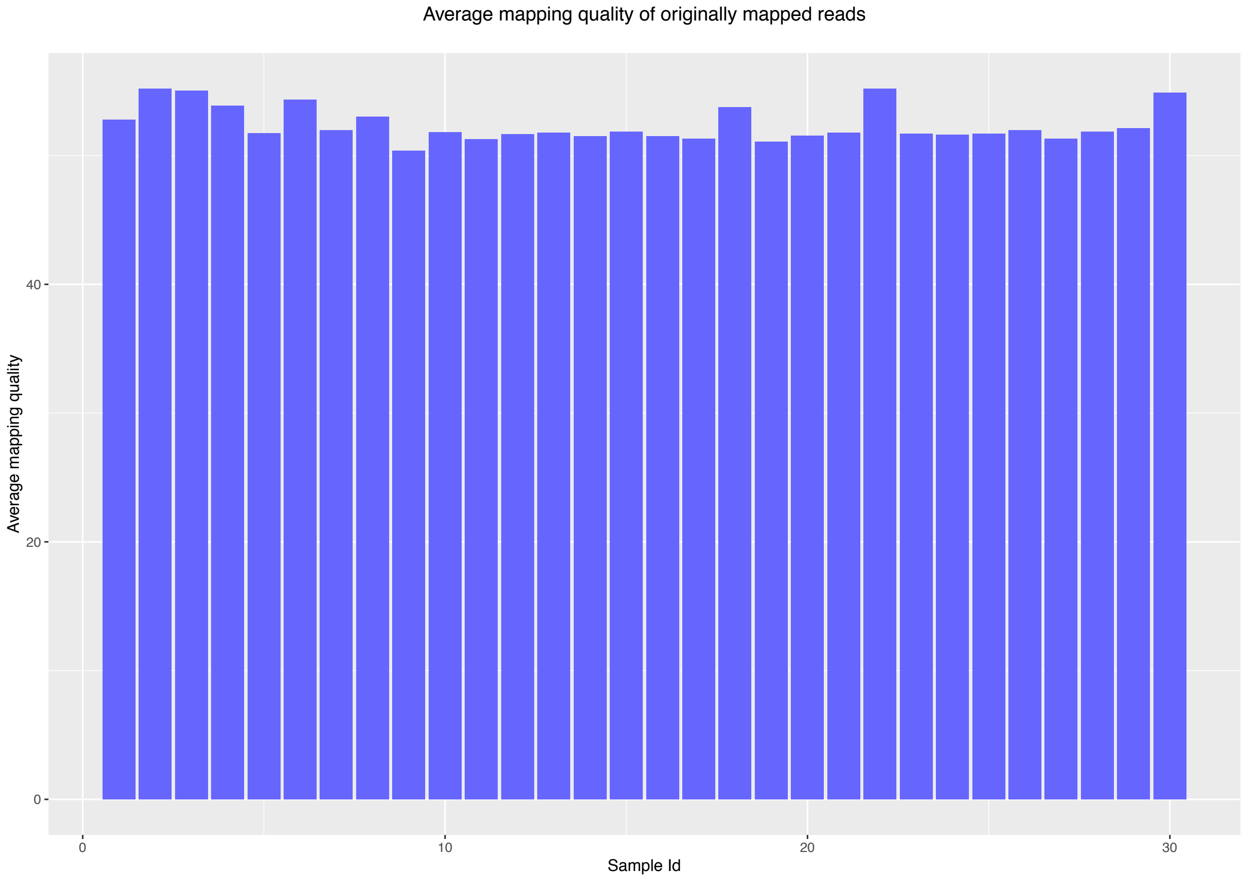

Supplementary Figure 5: Average mapping quality of the samples for the originally mapped reads.
